# Extra‐anatomic aortic bypass with aortic‐, mitral‐, and tricuspid surgery in a 53‐year old: A single‐stage approach for complex coarctation associated with triple valve pathology

**DOI:** 10.1111/jocs.14465

**Published:** 2020-02-17

**Authors:** Paul Werner, Daniel Zimpfer, Günther Laufer, Dominik Wiedemann

**Affiliations:** ^1^ Division of Cardiac Surgery, Department of Surgery Medical University of Vienna Vienna Austria

**Keywords:** aortic coarctation, extraanatomical aortic bypass, multiple valve pathology

## Abstract

Coarctation is rare in patients over 50 years of age; however, if present, it can be associated with complex intracardiac pathologies and represent a formidable surgical challenge. Herein, we report a single‐stage approach for surgical repair of coarctation associated with aortic, mitral, and tricuspid valve pathology using an ascending‐to‐descending aortic bypass with posterior pericardial access.

## 1 CASE REPORT

**Figure 1:**
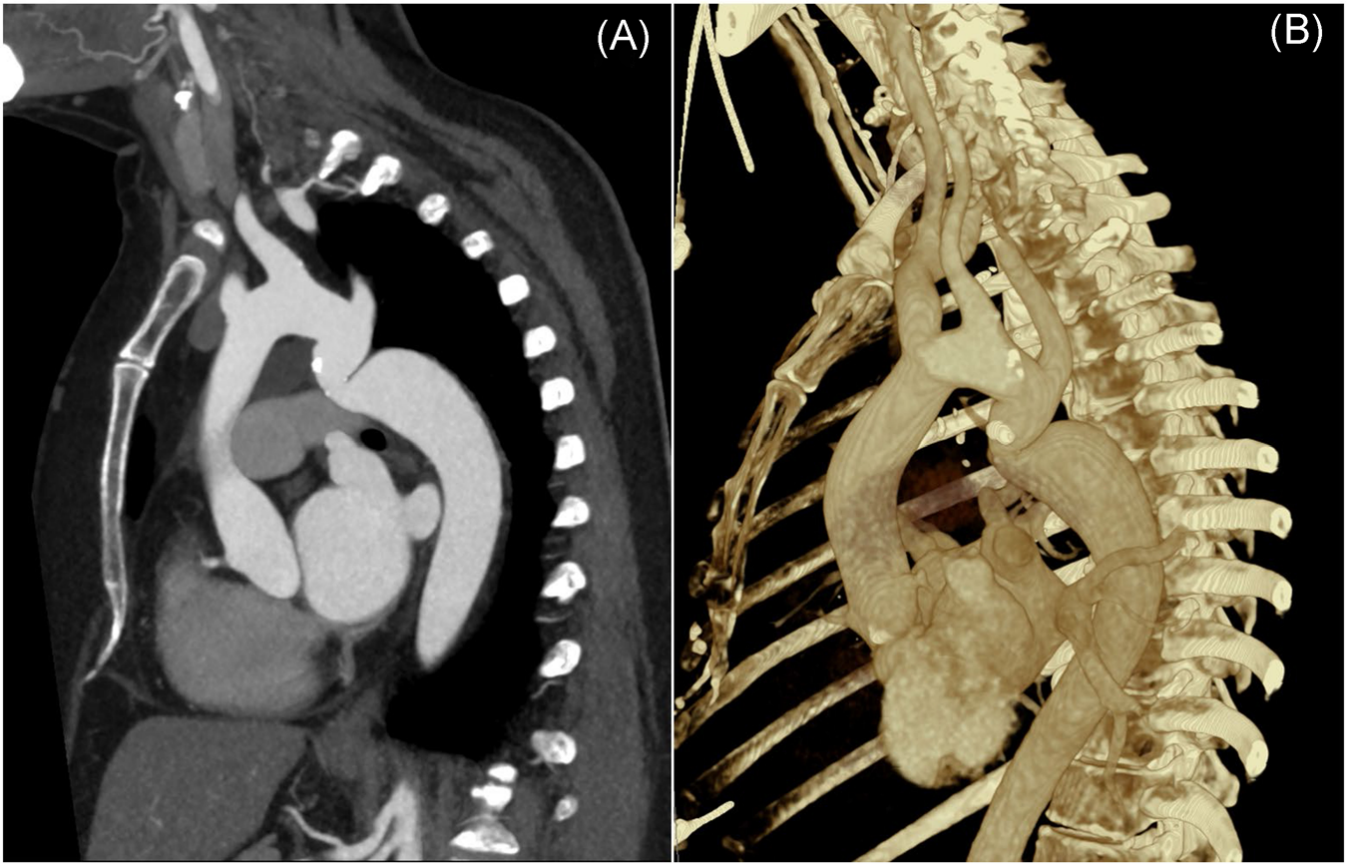
Preoperative computed tomography (CT) in sagittal axis showing aortic coarctation (A) native view (B) CT‐based three‐dimensional reconstruction

A 54‐year‐old female presented with dyspnea NYHA II based on multiple valve pathology with associated coarctation (Figure 1) and long‐standing hypertension, chronic obstructive pulmonary disease and depression. Transoesophageal echocardiography (TEE) revealed a stenotic bicuspid aortic valve (opening area 0.7 cm^2^) and highly insufficient mitral and tricuspid valves. The mean pulmonary‐artery and pulmonary‐capillary‐wedge‐pressure were 34 and 31 mm Hg, respectively. The coarctation showed a mean gradient of 33 mm Hg. Left ventricular function was preserved while the right‐ventricular function was moderately reduced. The calculated EuroSCORE II mortality was 4.85%.

### 1.1 Surgical technique

**Figure 2:**
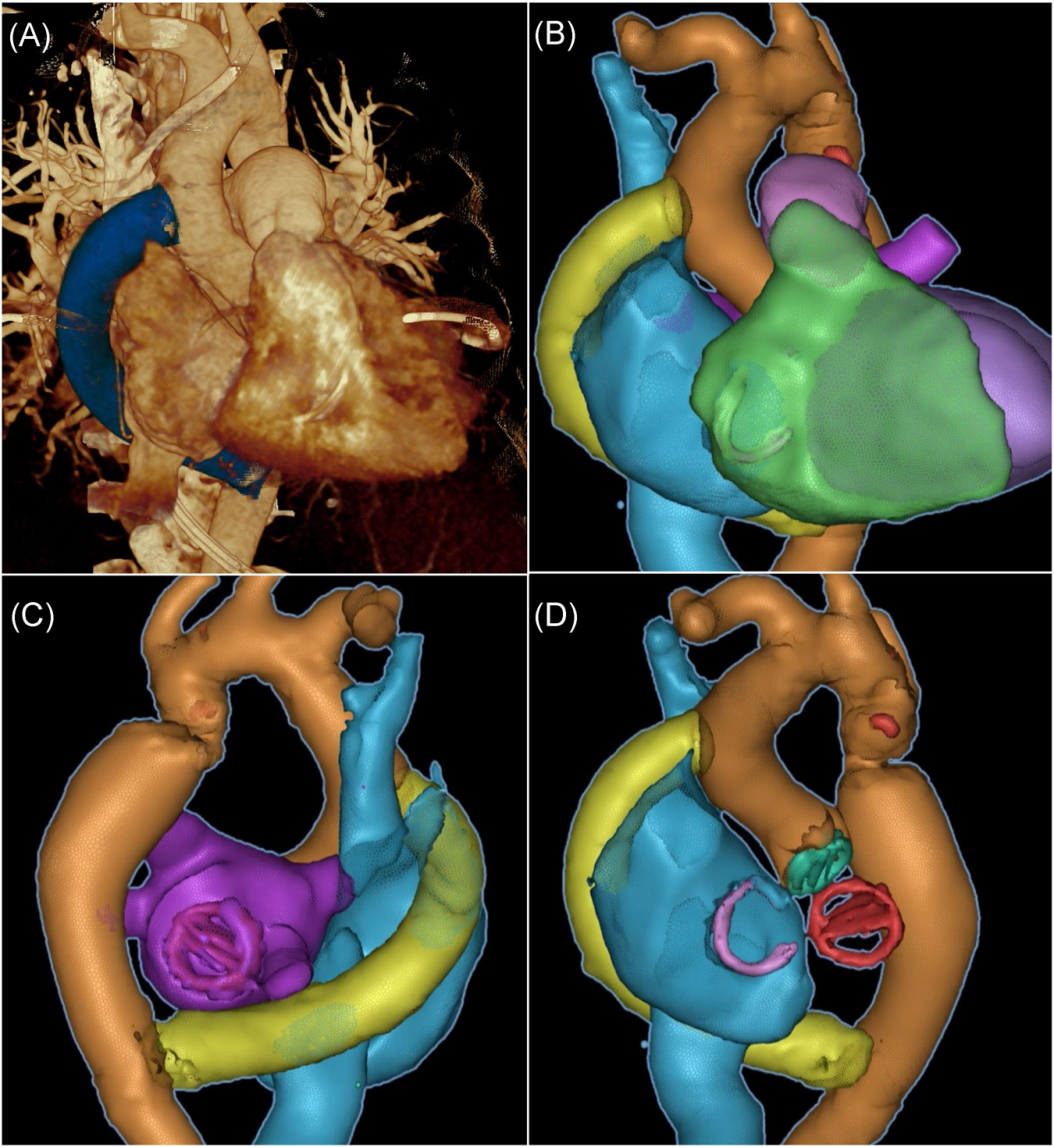
Postoperative computed tomography (CT) of ascending‐to‐descending bypass routed behind the inferior vena cava. (A) Three‐dimensional segmentation highlighting the bypass routing. (B) CT‐based reconstruction of ascending‐to‐descending bypass (yellow), cardiac structures and implants. (C) Aortic (green) and mitral valve (red) prostheses and tricuspid ring (purple). (D) Posterior view of ascending‐to‐descending bypass showing calcifications in the isthmus area (red)

Surgery was performed under general anesthesia with radial and femoral arterial monitoring. After median sternotomy, cardiopulmonary bypass (CPB; distal ascending aorta cannulation) with mild hypothermia (32°C) was initiated. The right‐sided pleura was opened for the displacement of the heart to expose the posterior pericardium, which was incised vertically below the left pulmonary veins. The descending aorta was partially clamped, with respect to the esophagus. The distal anastomosis of the ascending‐to‐descending bypass was performed using an 18‐mm polyester prosthesis (B. Braun, Hessen, Germany) and Prolene 4‐0. Individual sizing of the graft was performed by the surgeon after assessment of the ascending aortic diameter in the preoperative computed tomography angiography. After adequate hemostasis, the heart was repositioned, the ascending aorta was cross‐clamped, and the aortic valve was excised. The subvalvular inspection confirmed a septal bulge seen on intraoperative TEE, and extensive myectomy was performed. The left atrium was opened and the mitral valve presented as not repairable; therefore a 25/33‐mm On‐X prosthesis (CryoLife, GA) was implanted. Aortic valve replacement was performed using a 19‐mm On‐X prosthesis. After the release of the aortic cross‐clamp and opening of the right atrium (RA), a persistent foramen ovale (PFO) was found and suture closed. Subsequently, beating‐heart tricuspid annuloplasty using a 30‐mm Contour 3D ring (Medtronic, Leinster, Ireland) was performed. The aortic bypass graft was then tunneled behind the inferior vena cava (IVC) along the free RA wall to the ascending aorta where the proximal anastomosis was sewn onto the partially clamped aorta in an end‐to‐side fashion (Prolene 5‐0) (Figure 2). The patient was weaned from CPB and inhalative nitric oxide treatment was started due to elevated pulmonary pressures. She experienced a prolonged intensive care unit stay due to postoperative renal failure requiring intermittent hemodialysis until the 67^th^ postoperative day (POD) and complicated respiratory weaning (39 days on a respirator). She underwent pacemaker implantation due to a total AV block and was finally discharged in improved general condition on the 79^th^ POD. In the 5‐month follow‐up, she reported improved dyspnea NYHA I and normotensive systolic blood pressure (SBP) values with reduced single‐agent antihypertensive therapy.

## 2 DISCUSSION

To our knowledge, this case reports the first mechanical aortic and mitral valve replacement with concomitant tricuspid repair, PFO closure, left ventricular outflow tract myectomy and ascending‐to‐descending aortic bypass. In patients suffering from coarctation and associated intracardiac pathology, following treatment modalities do exist: two‐stage repair, single‐stage simultaneous repair, and hybrid interventions using endovascular techniques.^1^

The two‐stage repair can achieve anatomic correction of coarctation, nevertheless, it implies two separate surgical procedures. Correction of intracardiac pathology is generally preferred first, yet, in our case, the patient's heart would have been exposed to a considerable afterload of the coarctation following an extensive cardiac surgery with the risk of cardiac failure or severe hypertension. Conversely, initial coarctation repair bares the risk of perioperative cardiac decompensation due to uncorrected intracardiac pathologies.

Hybrid procedures with balloon dilation or stent implantation might present a safe alternative in younger/anatomically‐feasible patients. However, stent implantation in patients above 40 years of age, as the presented case, may lead to life‐threatening complications including aortic dissection or rupture.^2^

A single‐stage procedure with ascending‐to‐descending bypass offers several advantages. Treatment of all pathologies can be addressed within one intervention. Ascending‐to‐descending bypass for coarctation offers excellent long‐term results and low perioperative morbidity and mortality; Said et al^3^ reported on 80 patients (median age 42 years) with no early mortality, no incidence of paraplegia or stroke and a decrease of mean SBP from 153 ± 26 to 123 ± 15 mm Hg postoperatively. The posterior pericardial access to the descending aorta possesses a low risk for paraplegia, as no extensive aortic dissection and mobilization are required. Routing of the graft posterior to the IVC minimizes the risk of graft injury during a redo situation.

## 3 CONCLUSIONS

The present case highlights the importance of the ascending‐to‐descending bypass over median sternotomy for surgical coarctation treatment in adults with concomitant cardiac disease. Given a systematic surgical approach, this technique provides a single‐stage repair of coarctation and aortic, mitral, and tricuspid valve surgery with favorable outcomes.

## CONFLICT OF INTERESTS

Daniel Zimpfer is an advisory board member (Abbott, Medtronic, Berlin Heart), a proctor (Abbott, Medtronic), and received speaker fees (Abbott, Medtronic). Guenther Laufer is an advisory board member (Edwards). Dominik Wiedemann is a proctor (Abbott, Medtronic).
